# A common deletion at *BAK1* reduces enhancer activity and confers risk of intracranial germ cell tumors

**DOI:** 10.1038/s41467-022-32005-9

**Published:** 2022-08-02

**Authors:** Kyuto Sonehara, Yui Kimura, Yoshiko Nakano, Tatsuya Ozawa, Meiko Takahashi, Ken Suzuki, Takashi Fujii, Yuko Matsushita, Arata Tomiyama, Toshihiro Kishikawa, Kenichi Yamamoto, Tatsuhiko Naito, Tomonari Suzuki, Shigeru Yamaguchi, Tomoru Miwa, Hikaru Sasaki, Masashi Kitagawa, Naoyuki Ohe, Junya Fukai, Hideki Ogiwara, Atsufumi Kawamura, Satoru Miyawaki, Fumihiko Matsuda, Nobutaka Kiyokawa, Koichi Ichimura, Ryo Nishikawa, Yukinori Okada, Keita Terashima

**Affiliations:** 1 Department of Statistical Genetics, Osaka University Graduate School of Medicine, Suita, 565-0871 Japan; 2 Integrated Frontier Research for Medical Science Division, Institute for Open and Transdisciplinary Research Initiatives, Osaka University, Suita, 565-0871 Japan; 3 Division of Neuro-Oncology, Children’s Cancer Center, National Center for Child Health and Development, Tokyo, 157-8535 Japan; 4 Division of Brain Tumor Translational Research, National Cancer Center Research Institute, Tokyo, 104-0045 Japan; 5 Department of Pediatrics, The University of Tokyo Hospital, Tokyo, 113-8655 Japan; 6 Center for Genomic Medicine, Kyoto University Graduate School of Medicine, Kyoto, 606-8501 Japan; 7 Department of Neurosurgery, National Defense Medical College, 3-2 Namiki, Tokorozawa, Saitama, 359-8513 Japan; 8 Department of Neurosurgery, Juntendo University School of Medicine, 2-1-1 Hongo, Bunkyo-ku, Tokyo, 113-8421 Japan; 9 Department of Brain Disease Translational Research, Graduate School of Medicine, Juntendo University, 2-1-1 Hongo, Bunkyo-ku, Tokyo, 113-8421 Japan; 10 Department of Otorhinolaryngology - Head and Neck Surgery, Osaka University Graduate School of Medicine, Suita, 565-0871 Japan; 11 Department of Head and Neck Surgery, Aichi Cancer Center Hospital, Nagoya, 464-8681 Japan; 12 Department of Pediatrics, Osaka University Graduate School of Medicine, Suita, 565-0871 Japan; 13 Laboratory of Statistical Immunology, Immunology Frontier Research Center (WPI-IFReC), Osaka University, Suita, 565-0871 Japan; 14 Department of Neurology, Graduate School of Medicine, the University of Tokyo, Tokyo, 113-8655 Japan; 15 Department of Neuro-Oncology/Neurosurgery, Saitama Medical University International Medical Center, Hidaka, 350-1298 Japan; 16 Department of Neurosurgery, Faculty of Medicine, Hokkaido University, Sapporo, 060-8648 Japan; 17 Department of Neurosurgery, Keio University School of Medicine, Tokyo, 160-8582 Japan; 18 Department of Neurosurgery, Shizuoka Children’s Hospital, Shizuoka, 420-8660 Japan; 19 Department of Neurosurgery, Graduate School of Medicine, Gifu University, Gifu, 501-1194 Japan; 20 Department of Neurological Surgery, Wakayama Medical University School of Medicine, Kimiidera, 641-8509 Japan; 21 Division of Neurosurgery, National Center for Child Health and Development, Tokyo, 157-8535 Japan; 22 Department of Neurosurgery, Hyogo Prefectural Kobe Children’s Hospital, Kobe, 650-0047 Japan; 23 Department of Neurosurgery, Faculty of Medicine, the University of Tokyo, Tokyo, 113-8655 Japan; 24 Department of Pediatric Hematology and Oncology Research, National Center for Child Health and Development, Tokyo, 157-8535 Japan; 25 The Center for Infectious Disease Education and Research (CiDER), Osaka University, Suita, 565-0871 Japan; 26 Laboratory for Systems Genetics, RIKEN Center for Integrative Medical Sciences, Yokohama, 230-0045 Japan; 27 Department of Genome Informatics, Graduate School of Medicine, the University of Tokyo, Tokyo, 113-0033 Japan

**Keywords:** Cancer genetics, Epigenetics, Genome-wide association studies, Paediatric cancer

## Abstract

Intracranial germ cell tumors (IGCTs) are rare brain neoplasms that mainly occur in children and adolescents with a particularly high incidence in East Asian populations. Here, we conduct a genome-wide association study (GWAS) of 133 patients with IGCTs and 762 controls of Japanese ancestry. A common 4-bp deletion polymorphism in an enhancer adjacent to *BAK1* is significantly associated with the disease risk (rs3831846; *P* = 2.4 × 10^−9^, odds ratio = 2.46 [95% CI: 1.83–3.31], minor allele frequency = 0.43). Rs3831846 is in strong linkage disequilibrium with a testicular GCTs susceptibility variant rs210138. In-vitro reporter assays reveal rs3831846 to be a functional variant attenuating the enhancer activity, suggesting its contribution to IGCTs predisposition through altering *BAK1* expression. Risk alleles of testicular GCTs derived from the European GWAS show significant positive correlations in the effect sizes with the Japanese IGCTs GWAS (*P* = 1.3 × 10^−4^, Spearman’s *ρ* = 0.48). These results suggest the shared genetic susceptibility of GCTs beyond ethnicity and primary sites.

## Abstract (web summary)

Intracranial germ cell tumors (IGCTs) are rare brain tumors mainly diagnosed in children and young adults. Here, the authors conduct a genome-wide association study for IGCTs, identify a risk locus at *BAK1*, and characterize its functional consequences.

## Introduction

Germ cell tumors (GCTs) are a heterogeneous group of rare neoplasms that occur in the gonads (testes and ovaries) and also in extragonadal sites of the body (mediastinum, peritoneum, sacrum, and brain). GCTs localized in the brain are called intracranial germ cell tumors (IGCTs), which mainly arise in children and adolescents. IGCTs are histologically classified into two major groups: germinoma (the most frequent subtype of IGCTs) and non-germinomatous germ cell tumors (NGGCTs) including teratoma, yolk sac tumor, choriocarcinoma, and embryonal carcinoma. Germinoma is generally sensitive to radiotherapy and chemotherapy and shows good prognosis, whereas NGGCTs often exhibit resistance to treatment and poor prognosis^1^.

One of the characteristic features of IGCTs is their significant regional differences in incidence. It is substantially higher in East Asian countries than in Western countries (e.g., an incidence of 2.7/million/year in Japan but 0.6/million/year in the United States)^2^. This regional disparity is significant compared to all other brain tumors. In addition, although gonadal GCTs are histologically similar to IGCTs, they show the opposite trend in regional prevalence (e.g., testicular GCTs have an incidence of 55/million/year in the United States but 25/million/year in Japan)^3^.

The heterogeneity in histology and the striking geographical difference in epidemiology have attracted the interests of clinicians and researchers. However, only a limited amount of basic research on IGCTs has been conducted due to the low incidence and difficulty obtaining tumor specimens because of the frequent occurrence in the neurohypophysis and pineal region, where surgical resection is difficult. The biological basis of these tumors is still largely unknown.

Recently, rare germline variants in *JMJD1C*, a chromatin modifier involved in germinal tissue development, were implicated in IGCTs risk^4^. In contrast, the contribution of common variants to the risk of IGCTs has never been thoroughly investigated. Considering recent evidence that common genetic variation is involved in the susceptibility of other GCTs (e.g., testicular GCTs^5–7^ and pediatric GCTs^8^), we hypothesized that common variants should also contribute to IGCTs.

Here, we conduct an initial GWAS of IGCTs in the Japanese population with nationwide efforts to involve >130 patients, which offers an advantage in the scale compared to previous studies on IGCTs germline genetics. We perform whole-genome genotype imputation to fine-map the risk variant. In silico functional annotation using epigenome databases and in vitro reporter assays elucidate the causal mechanism of the risk variant. We further evaluate shared genetic predispositions between IGCTs and TGCTs.

## Results

### Genome-wide association study of IGCTs

We enrolled a total of 138 patients with intracranial germ cell tumors (IGCTs) and 808 healthy volunteers. After stringent quality control, 497,059 directly genotyped SNPs of 133 cases and 762 controls were included in the subsequent genotype imputation. Consistent with the general observation that the Japanese population is genetically homogeneous^9^, the principal component vectors of the genome-wide genotypes confirmed that the genetic ancestry of cases and controls were well matched (Supplementary Fig. 1). To extend the coverage of the genetic variants, we performed whole-genome genotype imputation using the combined reference panel of 1000 Genomes Project Phase 3 version 5 (1KG) genotype (*n* = 2504) and Japanese whole-genome sequencing data (*n* = 1037)^10,11^. We analyzed 8,308,293 autosomal variants and 222,270 X-chromosomal variants that fulfilled stringent post-imputation quality control criteria (minor allele frequency [MAF] > 0.5% and *Rsq* by Minimac3 > 0.7). The quantile-quantile plot of the association *P* values indicated little genomic inflation (genomic inflation factor [*λ*_GC_] 1.016).

**Fig. 1:**
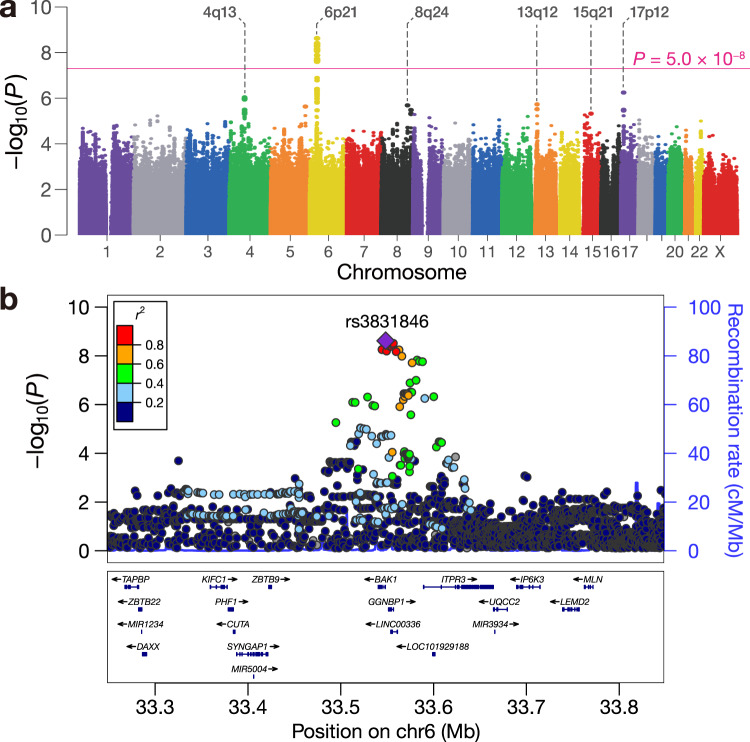
Genome-wide association study of intracranial germ cell tumors. **a** Genome-wide associations of imputed genetic variants are shown. The pink horizontal line indicates the genome-wide significance threshold of *P* = 5.0 × 10^−8^. The loci satisfying the suggestive significance threshold (*P* = 5.0 × 10^−6^) are annotated. **b** Regional associations of the imputed genetic variants at the genome-wide significant locus are shown. The purple diamond indicates the lead variant, rs3831846. Other circles are colored by LD (*r*^2^) with the lead variant based on the EAS individuals of the reference panel used for genotype imputation^10,11^. All statistical tests are two-sided and not adjusted for multiple comparisons.

We detected a genetic locus surpassing the genome-wide significance threshold at 6p21 (Fig. 1a). The genetic variant with the strongest association in the locus was rs3831846 (*P* = 2.4 × 10^−9^, OR 2.46 [95% CI: 1.83–3.31]; Fig. 1b; Table 1), which was located 270 bp upstream of the *BAK1* gene. The risk allele frequency of rs3831846 in the control participants (= 0.43) was comparable to that in the Japanese population of 1KG ( = 0.40) (Supplementary Fig. 2). Rs3831846 was in strong linkage disequilibrium (LD) with rs210138, the previously reported risk variant of testicular germ cell tumors (TGCTs)^5–7^ (*r*^2^ = 0.98 in both European [EUR] and East Asian [EAS] populations of 1KG). Rs210138 was directly genotyped in our study and also fulfilled the genome-wide significance threshold (*P* = 7.2 × 10^−9^, OR 2.39 [95% CI: 1.78–3.21]; Table 1). We performed conditioning analysis in two settings separately: (i) analysis adjusted for the genotype of rs3831846 (Supplementary Fig. 3a) and (ii) rs210138 (Supplementary Fig. 3b). In both settings, no additional association was observed in the locus, suggesting that the two associated variants represent the same association signal. Other than 6p21, we additionally identified five associated loci with suggestive significance (*P* < 5.0 × 10^−6^), including 4q13, 8q24, 13q12, 15q21, and 17p12 (Fig. 1a; Supplementary Table 1; Supplementary Fig. 4). The lead variant at 8q24 (rs56361736; *P* = 2.1 × 10^−6^, OR 3.28 [95% CI: 2.01–5.34]) is an intronic SNP of the *DEPTOR* gene, which was recently implicated in a European TGCTs GWAS^12^.

**Table 1:** Association results for the imputed and directly genotyped lead variants

SNP	Genotyping	Chr	Position (NCBI build 37)	Alleles	Risk Allele	Freq. Case	Freq. Ctrl	Odds Ratio (95% CI)	*P* value
rs3831846	Imputed	6	33,548,346	TGTAA/T	T	0.63	0.43	2.46 (1.83–3.31)	2.4 × 10^−9^
rs210138	Directly genotyped	6	33,542,538	A/G	G	0.63	0.43	2.39 (1.78–3.21)	7.2 × 10^−9^

Freq. Case, risk allele frequency in cases; Freq. Ctrl, risk allele frequency in controls. All statistical tests are two-sided and not adjusted for multiple comparisons.

**Fig. 2:**
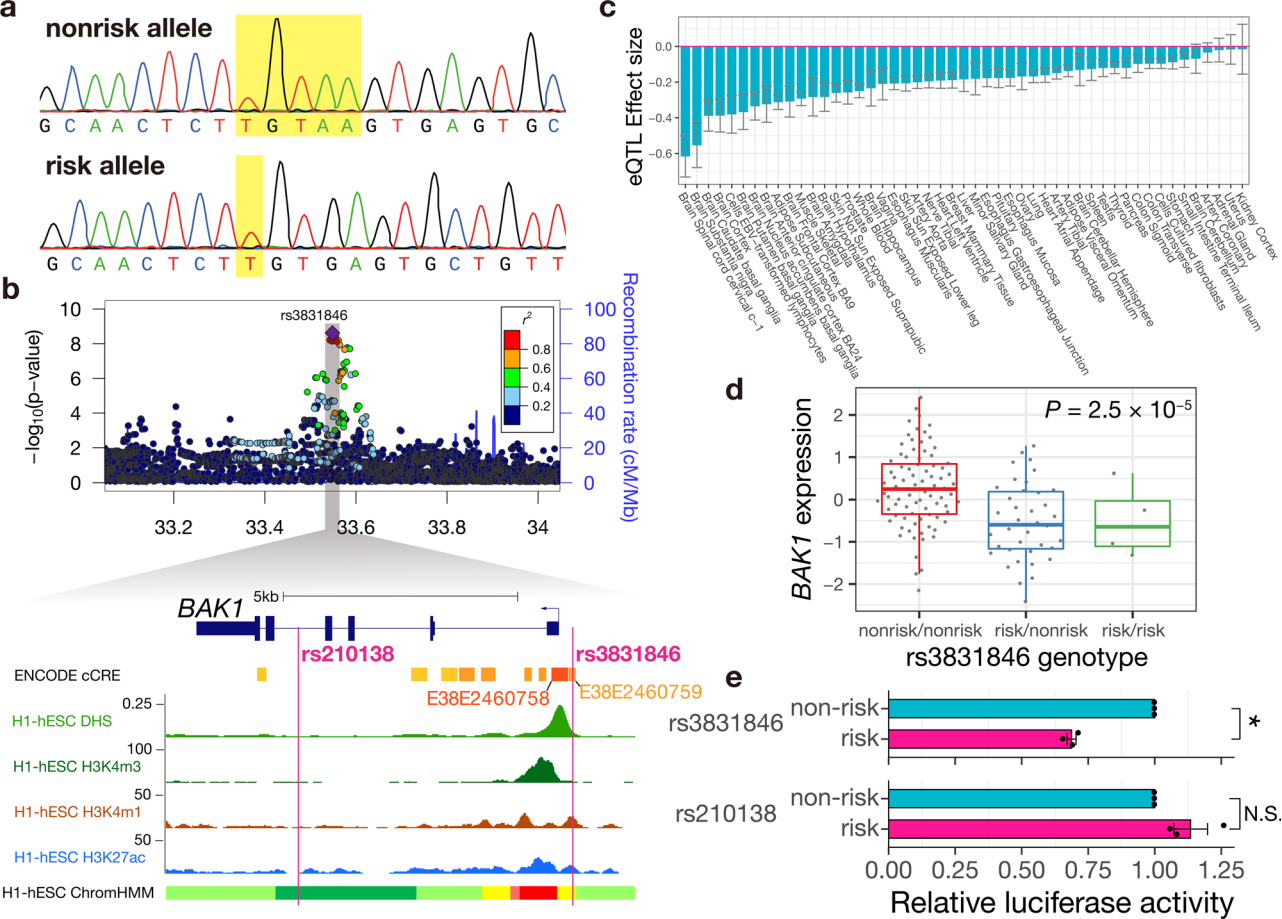
Functional characterization of the fine-mapped deletion polymorphism rs3831846. **a** The deletion polymorphism rs3831846 was validated by Sanger sequencing. The polymorphic site is highlighted in yellow. **b** Regional plot of imputed variants and epigenetic signatures in H1-hESC. The epigenetic signatures were obtained from the UCSC Genome Browser. E38E2460758 is a promoter-like signature. E38E2460759 is a proximal enhancer-like signature. cCRE, candidate *cis-*regulatory element; DHS DNase hypersensitive site. **c** The eQTL effect sizes of rs3831846 on the expression of *BAK1* for each tissue in the GTEx v8 dataset are shown. The center of error bars represents the point estimate of the effect size. Error bars represent S.E. Source data including the sample size of each tissue are provided as a Source Data file. **d** Representative association of rs3831846 genotype with *BAK1* expression (tissue: brain spinal cord cervical c-1). Each dot represents the normalized expression value of *BAK1* of each individual in the GTEx v8 dataset (*n* = 126 individuals). Boxplots represent the interquartile range (IQR), ends of whiskers represent minimum and maximum values within 1.5 × IQR. **e** Relative NanoLuc reporter activity to the *BAK1* enhancer (E38E2460759) sequence with either the risk allele or non-risk allele of rs3831846 (top) and the genomic sequence around rs210138 with either the risk or non-risk allele (bottom) is shown normalized to the Firefly luciferase activity in 293 T cells. The relative luciferase activity is normalized to the non-risk allele. The mean (SEM; error bars) of three independent experiments performed in technical triplicate is shown. *P = 2.9 × 10^−3^ by Welch’s *t*-test. N.S., *P* > 0.05 by Welch’s *t*-test. All statistical tests are two-sided and not adjusted for multiple comparisons.

The lead variant rs3831846 at 6p21 is a deletion polymorphism of four base pairs (non-risk allele, TGTAA; risk allele, T). Rs3831846 was computationally imputed based on the reference panel, which had been constructed using whole-genome sequencing^10,11^. Since array-based genotype imputation is relatively vulnerable to deletion polymorphisms, as technical validation of the imputation, we performed Sanger sequencing of rs3831846 of the 14 patients in the GWAS participants (Fig. 2a). We compared the Sanger sequencing-based genotypes with those imputed by array data and confirmed a high concordance rate of 100%. Next, as a replication analysis, we performed Sanger sequencing of rs3831846 of another 99 IGCTs patients from ref. 13 and observed a remarkably high risk allele frequency of 0.62 (95% CI: 0.55–0.69), which is comparable to that in the cases of the discovery GWAS dataset. We compared the allele frequency of the 99 patients with a control genotype dataset of a general Japanese population^10^ (*n* = 1026; risk allele frequency = 0.42), confirming rs3831846 to be significantly associated in this replication analysis (*P* = 1.7 × 10^−7^, OR 2.22 [95% CI: 1.63–3.03]). The 14 patients subjected to the technical validation and the 99 patients for the replication analysis were both previously studied for the mutational profiles of the tumor specimens^13^. Leveraging the mutational profiles, we tested the association between the rs3831846 genotypes and the patterns of somatic mutations (the *KIT* gene, MAPK pathway, and PI3K pathway), but no significant association was found (Supplementary Table 2).

### Functional characterization of the IGCTs risk locus

Rs3831846 resides in a candidate *cis-*regulatory element (cCRE) E38E2460759, a promoter-proximal enhancer-like element defined by the ENCODE project^14^ lying 270 bp upstream of the *BAK1* gene (Fig. 2b). Distinct enhancer signatures, such as histone H3K4 mono-methylation and histone H3K27 acetylation, were observed at rs3831846. The genome sequence around rs3831846 was also annotated as an enhancer by the ChromHMM 15-state model^15^. In addition, rs3831846 is located in the open chromatin region in TGCTs cell lines^12^ (Supplementary Fig. 5). These epigenetic signals strongly suggest the regulatory function of rs3831846, in contrast to the lack of the signatures for the previously implicated intronic SNP, rs210138.

To examine the regulatory effect of rs3831846 on *BAK1* expression, we performed expression quantitative trait locus (eQTL) analysis using the GTEx v8 dataset^16^, revealing the widespread eQTL effect of rs3831846 on *BAK1* expression (Fig. 2c). The risk allele down-regulated *BAK1* expression (Fig. 2d). Given that the strong LD between rs3831846 and rs210138 hampers discrimination of the regulatory effects of the two variants, we further performed reporter assays using plasmid vectors in which the genomic sequences around the associated variants were inserted upstream of the minimal promoter. We tested the allelic differences (i) between the risk and non-risk allele of rs3831846 and (ii) between the risk and non-risk allele of rs210138. Consistent with the eQTL analysis results, the risk allele of rs3831846 reduced reporter activity, suggesting that rs3831846 attenuates the enhancer activity (Fig. 2e). In contrast, the risk allele of rs210138 did not exhibit a down-regulating effect. Together with the epigenetic signatures, these results firmly support the causal role of rs3831846 in the etiology of IGCTs.

**Fig. 3:**
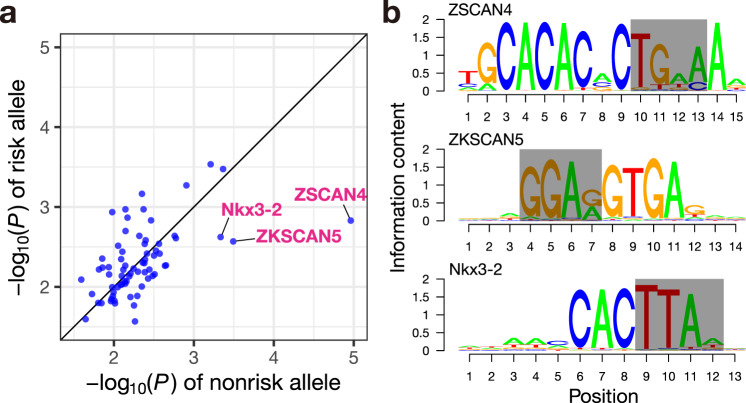
Disruption of transcription factor binding motifs by the deletion polymorphism rs3831846. **a** *P* values for sequence match of the risk allele or non-risk allele sequence with TF binding motifs are shown. Each dot indicates a TF binding motif. A smaller *P* value indicates a more significant sequence match with the TF binding motif. Note that we use the *P* value provided by PWMScan as an indicator of the extent to which the sequence and the TF binding motif match (Methods). Only the TF binding motifs whose binding score is calculated with the polymorphic site are shown. Multiple comparisons adjustments were not applied to the *P* values. **b** The three remarkably disrupted TF binding motifs. Grey boxes represent the bases deleted in the risk allele.

Given the essential role of transcription factor (TF) binding to enhancers for gene regulation^17,18^, we investigated the disrupting impact of the deletion polymorphism rs3831846 on the TF binding motifs within the enhancer element. Based on 746 TF binding profiles stored in the JASPAR database^19^, we assessed the differences in binding scores calculated by PWMScan^20^ between the risk and non-risk allele. We found that three TF binding motifs, ZSCAN4, ZKSCAN5, and Nkx3-2, exhibited an outstanding decrease in binding score by introducing the deletion (Fig. 3). These TFs may serve as potential candidates mediating the down-regulation of the *BAK1* expression for further in-depth analyses.

### Shared genetic background between IGCTs and TGCTs

**Fig. 4:**
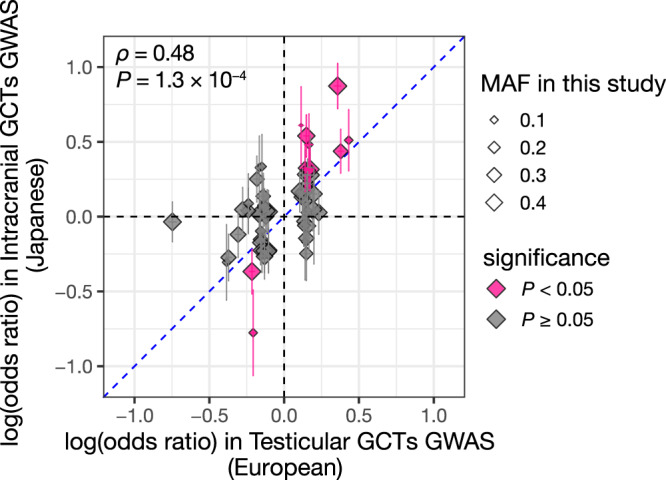
Comparison of the genetic risk of intracranial GCTs and testicular GCTs. Effect sizes in the previous TGCTs GWAS are compared with those in the IGCTs GWAS. Each marker represents a risk locus derived from the TGCTs GWAS. Effect size and S.E. are shown. The blue dashed line is the identity line. Marker sizes are proportional to MAF in this study. Pink markers indicate the 11 loci meeting nominal significance (*P* < 0.05) in the IGCTs GWAS. The *P* value is provided by the two-sided Spearman’s rank correlation test.

The observation that the IGCTs risk variant rs3831846 and the TGCTs risk variant rs210138 were in strong LD prompted us to comprehensively examine the published TGCTs GWAS results in the EUR population. Of the 66 TGCTs risk loci reaching genome-wide significance in the recently published large-scale TGCTs GWAS^12^, 57 associations were available in our study as the same or proxy common variant (Supplementary Data 1; see Methods). The effect sizes showed significantly positive overall correlations between IGCTs and TGCTs (*P* = 1.3 × 10^−4^, Spearman’s *ρ* = 0.48; Fig. 4). Notably, 11 loci exhibited nominally significant (*P* < 0.05) association with IGCTs: *CLPTM1L*, *PITX1*, *SPRY4*, *TNXB*, two loci of *BAK1*, *KATNA1*, *DEPTOR*, *GAB2-NARS2*, *HNF1B*, and *TKTL2* (Fig. 4; Supplementary Data 1). All the 11 loci showed the same effect direction with TGCTs GWAS (*P* = 9.8 × 10^−4^, sign test). These findings indicate the shared genetic background of the two types of GCTs beyond ethnicity and tumor location. Although the other 46 TGCTs risk loci were not significantly associated with IGCTs, the lack of significance could be potentially due to limited statistical power rather than the absence of the variant effect in IGCTs etiology. To assess whether the lack of significant association could be attributable to the statistical power issue, we performed a power calculation based on the odds ratios reported by the European TGCTs GWAS. The statistical power for most of the non-significant TGCTs risk variants (42 out of 46 variants [91%]) was less than 0.50 (Supplementary Fig. 6). We note that these significant and non-significant TGCTs risk variants did not show a systematic difference in the MAF in our study (*P* = 0.71; the Wilcoxon rank-sum test; Supplementary Fig. 7), suggesting that the lack of significance was not mainly due to low MAF in the Japanese population.

## Discussion

In this study, we demonstrated the significant contribution of common genetic variation to susceptibility to IGCTs. The most strongly associated variant was rs3831846, a deletion of four base pairs lying 270 bp upstream of *BAK1*. This deletion polymorphism resides in an enhancer region adjacent to the *BAK1* promoter. The in silico and in vitro regulatory analysis strongly suggested the functional role of the variant on decreased *BAK1* expression through disruption of TF binding motifs in the enhancer. Rs3831846 is in strong LD with rs210138, a SNP associated with the risk of TGCTs, which implies a shared causal effect of this locus on both types of GCTs. *BAK1* encodes a pro-apoptotic protein localized to mitochondria. This protein induces apoptosis by mitochondrial outer membrane permeabilization and resulting release of cytochrome c from mitochondria into the cytosol. Expression of *BAK1* is repressed by the KIT/KITLG pathway^21^, which plays a pivotal role in the survival of primordial germ cells (PGCs), the origin of GCTs^22,23^. At the stage of fetal development, PGCs migrate across the embryo from the yolk sac toward the gonads along the sympathetic nerve fibers. In this process, some PGCs may pass through the gonads, keep on the migration route along the midline of the body, and then reach other organs including the brain^24^. Those mis-migrated PGCs are to be removed by apoptosis in response to loss of the KIT/KITLG signal. The down-regulated *BAK1* expression may allow those mis-migrated PGCs to escape the removal and form GCTs^25^.

One of the unique characteristics of IGCTs is their remarkable geographical and ethnic difference in incidence^2^. Specifically, the incidence of IGCTs is approximately fourfold greater in EAS than EUR. The risk allele frequency of rs3831846 is higher in EAS than EUR (EAS 0.49, EUR 0.20 in 1KG), which may provide a partial explanation for the ethnic difference.

Comparison of the Japanese IGCTs GWAS and the European TGCTs GWAS proved the strong overall correlations in effect sizes. Moreover, our IGCTs GWAS demonstrated the 11 TGCTs risk loci associations with concordant risk alleles. Notably, these loci were implicated in a broad range of biological pathways, including KIT/KITLG signaling (*BAK1* and *SPRY4*), apoptosis regulation (*CLPTM1L*), and telomerase activity (*PITX1*). These findings provide evidence of shared genetic etiology of the two histologically similar tumors beyond ethnicity and tumor location, not limited to a specific biological pathway. The shared genetic etiology suggests the feasibility of trans-ethnic cross-GCTs genetic analysis, which will facilitate pinpointing true causal variants of GCTs by leveraging the trans-ethnic differences in patterns of LD^26^. We note that estimating the trans-ethnic genetic correlation based on the genome-wide entire associations^27,28^ should serve as another line of evidence of the shared genetic etiology. However, this approach generally requires more than thousands of the sample size for reliable estimation, which was regrettably not applicable to the current study and left for future work.

Given the frequent somatic mutations of the KIT/KITLG pathway in IGCTs^4,13^, one intriguing finding is the lack of association of rs4474514, the strongest TGCTs risk variant with an odds ratio of 2.11 at the *KITLG* locus. The statistical power analysis indicates that it is unlikely to be due to limited power (Supplementary Fig. 6). If the odds ratio is >1.46, the association of rs4474514 should be detected at the nominal significance threshold (*α* = 0.05) with a power of ~1.0, suggesting a weaker effect of rs4474514 for the Japanese IGCTs (Supplementary Fig. 8). This difference may be explained by the difference in the diseases (i.e., TGCTs and IGCTs) or the study populations (i.e., European and East Asian). We should also consider that the LD between rs4474514 and the true causal variant may differ between the East Asian and European populations. Since neither East Asian TGCTs GWAS nor European IGCTs GWAS is currently available, further work is warranted to understand the differential odds ratio.

In conclusion, our initial IGCTs GWAS revealed the genetic architecture of IGCTs, including similarities to that of TGCTs. Our findings demonstrate the feasibility of cross-GCTs genetic analysis. It will facilitate trans-ethnic meta-analysis with adequate sample size and improve fine-mapping of causal variants. Given that differences in molecular pathogenesis lies among histological subtypes of GCTs^29^, future work should include not only tumor-location-specific but also histological subtype-specific GWAS, which may lead to a more detailed description of the etiology of GCTs.

## Methods

### Study design and participants

Children and adults diagnosed with IGCTs were identified at the National Center for Child Health and Development and seven other recruiting hospitals throughout Japan and invited to participate in this study (*n* = 138). Patients and survivors were eligible for the study if they had a primary diagnosis of IGCTs including germinoma, embryonal carcinoma, yolk sac tumor, choriocarcinoma, teratoma, and mixed GCTs in the central nervous system^30^. Of the 138 patients, the date of diagnosis was available for 117. Thirty-four were incidental cases (identified within 1–2 years of diagnosis), and 83 were prevalent cases (identified over 2 years from diagnosis).

Healthy volunteers were recruited as controls from the Osaka University Graduate School of Medicine, the University of Tokyo, and affiliated institutes (*n* = 808). The control group also included genomic DNA from Epstein-Barr virus-transformed B-lymphoblast cell lines of unrelated Japanese individuals established by the Japan Biological Informatics Consortium. Of the 762 controls that passed quality control criteria described later, 758 (99.5%) were older than the median age at diagnosis of the cases (i.e., 16 years old). Although we did not necessarily confirm that all the controls were cancer-free, the control group did not include cancer cohorts.

All participants provided written informed consent with documents approved by the institutional review board of each participating institution. This study was approved by the ethical committee of the National Center for Child Health and Development and Osaka University.

### Genotyping, quality control, and genotype imputation

We genotyped 138 patients with IGCTs and 808 healthy volunteers using Infinium Asian Screening Array (Illumina, San Diego, CA, USA). This genotyping array was built using an East Asian reference panel including whole-genome sequences, which enabled effective genotyping in East Asian populations^31^. We performed genotype calling using GenomeStudio version 2.0.4 (Illumina, San Diego, CA, USA).

We applied stringent quality control filters to the genotyping dataset using PLINK version 1.90b4.4^32^ as described elsewhere^33^. We excluded individuals with a genotyping call rate <0.97. For pairs of closely related individuals (PI_HAT calculated by PLINK > 0.17), we removed the individuals with the lower call rate. We included only the individuals of the estimated East Asian ancestry, based on the principal component analysis with the individuals of the HapMap project^34^ using EIGENSOFT version 6.1.4^35^. We further excluded SNPs with (i) call rate <0.99, (ii) minor allele count <5, and (iii) *P* value for Hardy–Weinberg equilibrium <1.0 × 10^−5^ in controls. After applying quality control filters, we computed the top 20 principal components (PCs). Although we confirmed that the distribution of the PCs did not exhibit substantial differences between cases and controls (adjusted *P* > 0.05 for all the PCs by the Wilcoxon rank-sum test), to robustly correct for potential population stratification, we included the 20 PCs into the regression model as covariates in the subsequent association analysis.

We performed genome-wide genotype imputation to estimate untyped variants computationally. We used the combined reference panel of 1000 Genomes Project Phase 3 version 5 genotype (*n* = 2504) and Japanese whole-genome sequencing data (*n* = 1037)^10,11^ as a haplotype reference for genotype imputation. First, we excluded SNPs with >10% allele frequency difference with the representative reference datasets of Japanese ancestry, namely the combined reference panel aforementioned^10,11^ and the allele frequency panel of Tohoku Medical Megabank Project^36^. Second, we conducted haplotype estimation to improve imputation performance using SHAPEIT software version 2.r904^37^ with haplotype reference. After the prephasing, we used Minimac3 software version 2.0.1^38^ for genotype imputation. For the variants of the X chromosome, we performed prephasing and imputation separately for females and males. We also applied extensive quality control criteria to filter out the poorly imputed genetic variants. The variants imputed with *Rsq* >0.7 and a minor allele frequency >0.5% were used for the downstream analysis.

### Sanger sequencing of rs3831846

We performed Sanger sequencing of rs3831846 on two sets of individuals for the respective purposes: (i) 14 IGCTs patients in the GWAS for technical validation of the genotype imputation and (ii) 99 patients independent of the GWAS for replication analysis. Both sets of patients (i.e., a total of 113 patients) were included in the previous study^13^. DNA was extracted from frozen samples or blood using a DNeasy^®^ Blood and Tissue kit (QIAGEN). Genomic DNA was amplified using the following primers: Forward: 5’-GCTTTTCCCATCCCTGATTCTC-3’, Reverse: 5’-CCAATGCGACTACAGAACTG-3’. PCR products were sequenced using the forward PCR primer on ABI PRISM 3130xl Genetic Analyzer (Life Technologies, Applied Biosystems) with Big Dye Terminator v.3.1 Cycle Sequencing Kit (Life Technologies, Applied Biosystems) following the manufacturer’s instruction.

### Association analysis

We performed a genome-wide association test of the risk of IGCTs using a logistic regression model under the assumption of additive allelic effects of the variant dosages using PLINK2 version 2.00a3LM^39^. We set a genome-wide significance as *P* < 5.0 × 10^−8^ and a suggestive significance as *P* < 5.0 × 10^−6^. In reporting the loci with suggestive significance, we included only the variants with MAF > 1% in both cases and controls to make the findings more robust. We incorporated the top 20 principal components into the regression model as covariates to account for population stratification. For the variants of the X chromosome, we performed association tests separately for females and males and then meta-analyzed association results with the inverse-variance approach using METASOFT version 2.0.0^40^. Given that the risk locus at 6p21 was in the immediate vicinity of the major histocompatibility complex region, we conducted HLA imputation analysis^41^ using the population-specific reference panel of Japanese^42^, confirming no association of the HLA variants (Supplementary Fig. 9).

### Replication analysis

Of the 113 patients of the previous study^13^ on which we performed Sanger sequencing, we confirmed that 99 patients were not included in the discovery GWAS, and we considered these patients as the cases for replication analysis. As the controls, we derived the rs3831846 genotypes from whole-genome sequencing data of a general Japanese population (*n* = 1026)^10^ collected by the BioBank Japan Project. We assessed replication of the rs3831846 association by Fisher’s exact test.

### Expression quantitative trait locus (eQTL) analysis

We performed eQTL analysis using the lm() function implemented in R statistical software. We used the GTEx v8^16^ gene expression data (‘GTEx_Analysis_v8_eQTL_expression_matrices.tar’) and covariates data (‘GTEx_Analysis_v8_eQTL_covariates.tar.gz’) obtained from the GTEx portal. The genotype data of rs3831846 was downloaded via dbGaP (phs000424.v8.p2).

### Luciferase reporter assay

We generated *BAK1* enhancer reporter constructs (E38E2460759 defined by ENCODE 3^14^; rs3831846 risk allele or non-risk allele) by PCR-amplifying the pGEM-T easy vectors subcloned with the PCR products amplified using the forward primer AGCTGGTACCGCCCAGAACTGATGA (KpnI site underlined) and reverse primer AGCTGATATCCAGGGTGAGAAG (EcoRV site underlined). Similarly, we also generated reporter constructs including the rs210138 risk allele or non-risk allele using the forward primer AGCTGGTACCTTGGGTGCAAATCCAAGC (KpnI site underlined) and reverse primer GCTGATATCACACTGACTTCCCTAACTCTG (EcoRV site underlined). Then, the fragments were inserted into the pNL3.2 vector between the KpnI and EcoRV restriction sites. pNL3.2[NlucP/minP] (N104A), pGL4.53[luc2/PGK] (E501A), and pGEM-T easy (A1360) vectors were purchased from Promega.

293 T cells (ATCC: CRL-3216) were maintained according to the manufacturer’s protocol and were seeded at a density of 5 × 10^5^ cells in a 6 well format the day before transfection. These cells were used under 15 passages for preventing genotypic and phenotypic drift and authenticated using morphology/phenotypes with careful monitoring by our lab. Cells were then co-transfected pGL4.53[luc2/PGK] (control vector) and pNL3.2 (test vector) vector with 1:9 ratio (total 1 μg) using X-treamGENE9 transfection reagent (Roche) in 2 mL/well of culture medium. After 24 h of the transfection, cells were lysed with the Passive Lysis Buffer (Promega E1941) of 500 μL/well, and the lysates of 80 μL/well were transferred in white 96 well plates in triplicate. We then measured luciferase activity using the Nano-Glo Dual-Luciferase Reporter Assay System (Promega N1630) on a GloMax Explorer luminometer (Promega) according to the manufacturer’s protocol. Relative luciferase activity was calculated as the ratio of NanoLuc normalized to Firefly luciferase and non-risk allele control cells.

### Transcription factor binding site disruption analysis

We assessed the potential disruption of transcription factor binding sites by the lead variant. We extracted the sequence of the proximal enhancer-like element E38E2460759 defined by ENCODE 3. The element contains the lead GWAS variant rs3831846. To assess the impact of the variant on the transcription factor binding motifs, we fed the E38E2460759 sequence with the reference allele of rs3831846 and that with the alternative allele into PWMScan software version 1.1.9^20^ with the position weight matrix (PWM) library of vertebrates from JASPAR 2020^19^. Briefly, PWMScan evaluates provided sequence for matches to user-supplied PWMs and calculates the *P* value under the null hypothesis that the provided sequence is a random sequence of the given length and base composition. Our main focus is the difference between reference and alternative allele, but not the statistical significance itself for each sequence match. We used the *P* values as an indicator of the extent to which the E38E2460759 sequence and each TF binding motif match.

### Comparison with the testicular germ cell tumors GWAS

Referring to the previously published TGCTs GWAS of European ancestry (*n* = 189,839)^12^, we extracted the 66 independent lead variants with genome-wide significance (*P* < 5.0 × 10^−8^). For each TGCTs risk variant, we extracted the statistics of the same variant in the IGCTs GWAS and compared effect sizes if it was a common variant in our study (MAF > 0.05). When a lead variant in the TGCTs GWAS was not tested in our study, a proxy common variant with the highest LD (*r*^2^ > 0.8 in the European populations of the 1000 Genomes Project Phase 3 version 5) was alternatively assessed. Allele coding was harmonized between the studies so that the minor allele in our study was defined as the effect allele. The effect direction of the proxy variants was determined based on the in-phase allele pair. We estimated statistical power for each TGCTs risk variant in the IGCTs GWAS data to achieve the nominal significance threshold (*α* = 0.05) using the CaTS power calculator^43^.

### Reporting summary

Further information on research design is available in the Nature Research Reporting Summary linked to this article.

## Supplementary information

Supplementary Information

Description of Additional Supplementary Files

Supplementary Data 1

Reporting Summary

## Data Availability

The summary statistics of the GWAS results has been deposited in the National Bioscience Database Center (NBDC) Human Database (https://humandbs.biosciencedbc.jp/en/) under the accession number of hum0197 (https://humandbs.biosciencedbc.jp/en/hum0197-latest). Data can also be browsed at our pheweb.jp^44^ website (https://pheweb.jp/). GTEx v8 data was accessed via dbGaP study accession phs000424.v8.p2. The position weight matrix library of vertebrates from JASPAR 2020 was accessed at https://jaspar2020.genereg.net/download/data/2020/CORE/JASPAR2020_CORE_vertebrates_redundant_pfms_meme.zip. Source data for Fig. 2c are provided with this paper.

## Source data

Source Data
