# Supplementary material for: A common deletion at BAK1 reduces enhancer activity and confers risk of intracranial germ cell tumors
Source: Nat Commun. 2022 Aug 2;13:4478. doi: 10.1038/s41467-022-32005-9 (PMC9346128; doi:10.1038/s41467-022-32005-9)
Supplement: Supplementary file 1 — Supplementary Information [file 41467_2022_32005_MOESM1_ESM.pdf]

Supplementary Information for

A common deletion at *BAK1* reduces the enhancer activity and confers risk in intracranial germ cell tumors

Corresponding to Yukinori Okada (yokada@sg.med.osaka-u.ac.jp) and

Keita Terashima (terashima-k@ncchd.go.jp)

Table of Contents

Supplementary Figures ···· **3**

Supplementary Figure 1 | Visualization of the principal component vectors of the GWAS participants ···· 3

Supplementary Figure 2 | Allele frequency spectra of the risk allele of rs3831846 ···· 5

Supplementary Figure 3 | Conditioning analysis performed separately for rs3831846 and rs210138 ···· 6

Supplementary Figure 4 | Regional plots of additional five loci with suggestive significance ···· 7

Supplementary Figure 5 | Open chromatin regions at the *BAK1* locus in TGCT cell lines ···· 8

Supplementary Figure 6 | Estimated statistical power for the TGCTs risk variants in the IGCTs GWAS ···· 9

Supplementary Figure 7 | Minor allele frequency of the TGCTs risk variants in the IGCTs GWAS ···· 10

Supplementary Figure 8 | Estimated statistical power for the *KITLG* locus ···· 11

Supplementary Figure 9 | No evidence for the HLA variant associations ···· 12

Supplementary Tables ···· **13**

Supplementary Table 1 | Association results for the lead variants of the suggestive significant loci ···· 13

Supplementary Table 2 | Association test between rs3831846 and somatic mutational profiles ···· 14

Supplementary Data 1 is provided as a separate .xlsx file.

Supplementary Figures

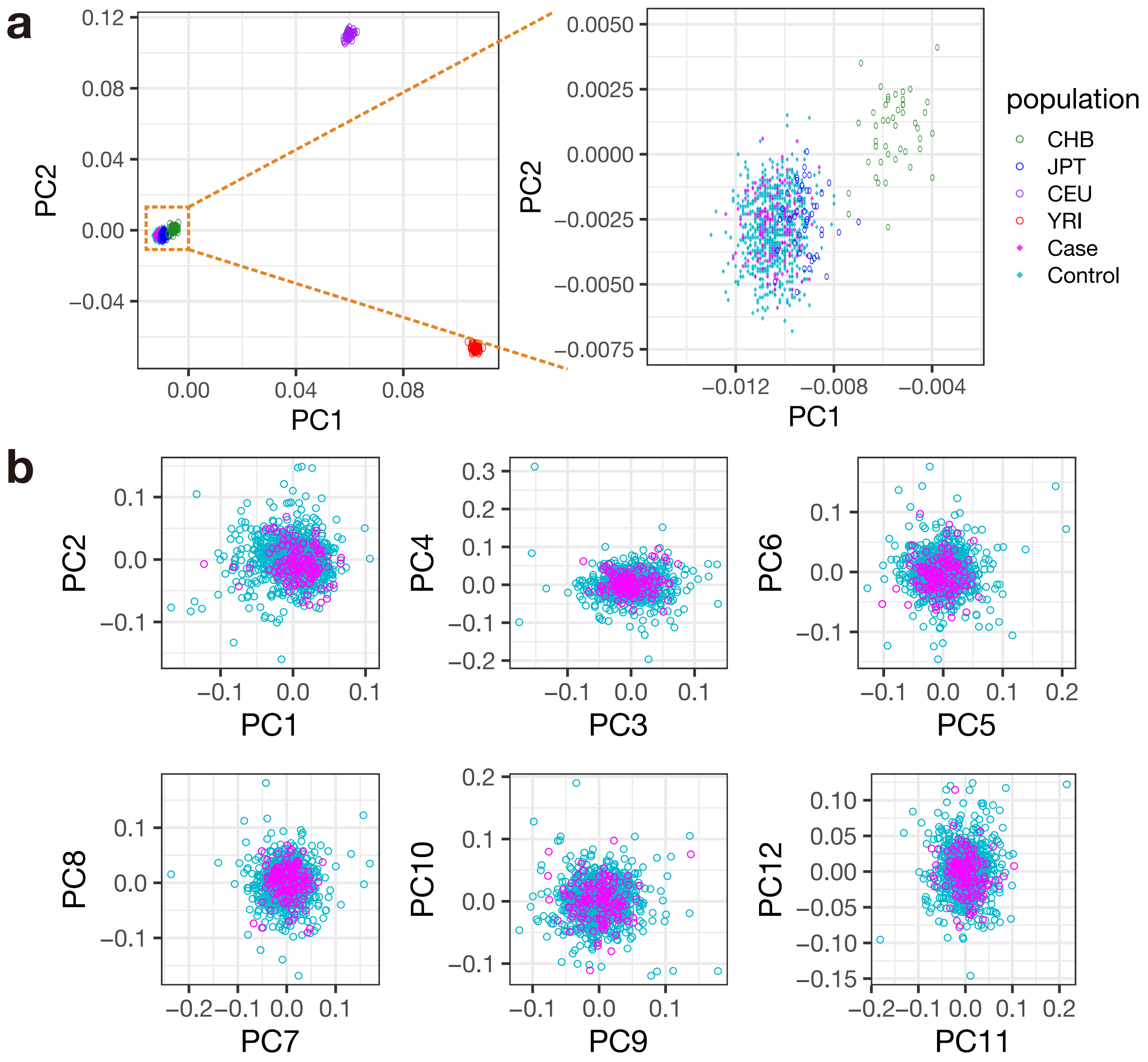

Supplementary Figure 1 | Visualization of the principal component vectors of the GWAS participants

(a) The first two principal component vectors of the genotypes of the study participants are indicated together with the individuals of the HapMap project. Each marker represents an

individual. CHB, Han Chinese in Beijing, China; JPT, Japanese in Tokyo, Japan; CEU, Utah residents with Northern and Western European ancestry; YRI, Yoruba in Ibadan, Nigeria. (**b)** The first 12 principal component vectors of the genome-wide genotypes of the study participants are plotted. Magenta markers indicate IGCTs cases (n = 133), and cyan markers do healthy controls (n = 762).

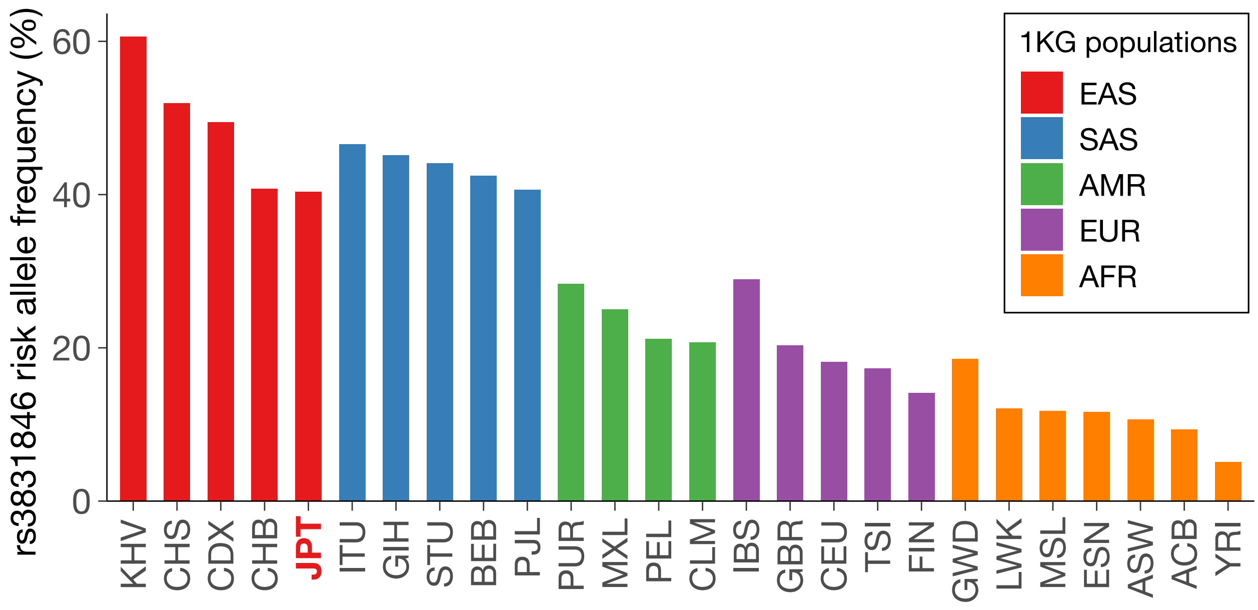

Supplementary Figure 2 | Allele frequency spectra of the risk allele of rs3831846

Allele frequencies of the risk allele of rs3831846 in the 1000 Genomes Project Phase 3 version 5 data are shown separately for each population. EAS, East Asian; SAS, South Asian; AMR, Ad Mixed American; EUR, European; AFR, African; JPT, Japanese in Tokyo, Japan; KHV, Kinh in Ho Chi Minh City, Vietnam; CHS, Southern Han Chinese; CDX, Chinese Dai in Xishuanagbanna, China; CHB, Han Chinese in Beijing, China; JPT, Japanese in Tokyo, Japan; ITU, Indian Telugu from the UK; GIH, Gujarati Indian from Houston, Texas; STU, Sri Lankan Tamil from the UK; BEB, Bengali from Bangladesh; PJL, Punjabi from Lahore, Pakistan; PUR, Puerto Rican from Puerto Rica; MXL, Mexican Ancestry from Los Angeles USA; PEL, Peruvian from Lima, Peru; CLM, Colombian from Medellian, Colombia; IBS, Iberian population in Spain; GBR, British in England and Scotland; CEU, Utah Residents (CEPH) with Northern and Western European ancestry; TSI, Toscani in Italia; FIN, Finnish in Finland; GWD, Gambian in Western Division – Mandinka; LWK, Luhya in Webuye, Kenya; MSL, Mende in Sierra Leone; ESN, Esan in Nigera; ASW, American's of African Ancestry in SW USA; ACB, African Carribean in Barbados; YRI, Yoruba in Ibadan, Nigera.

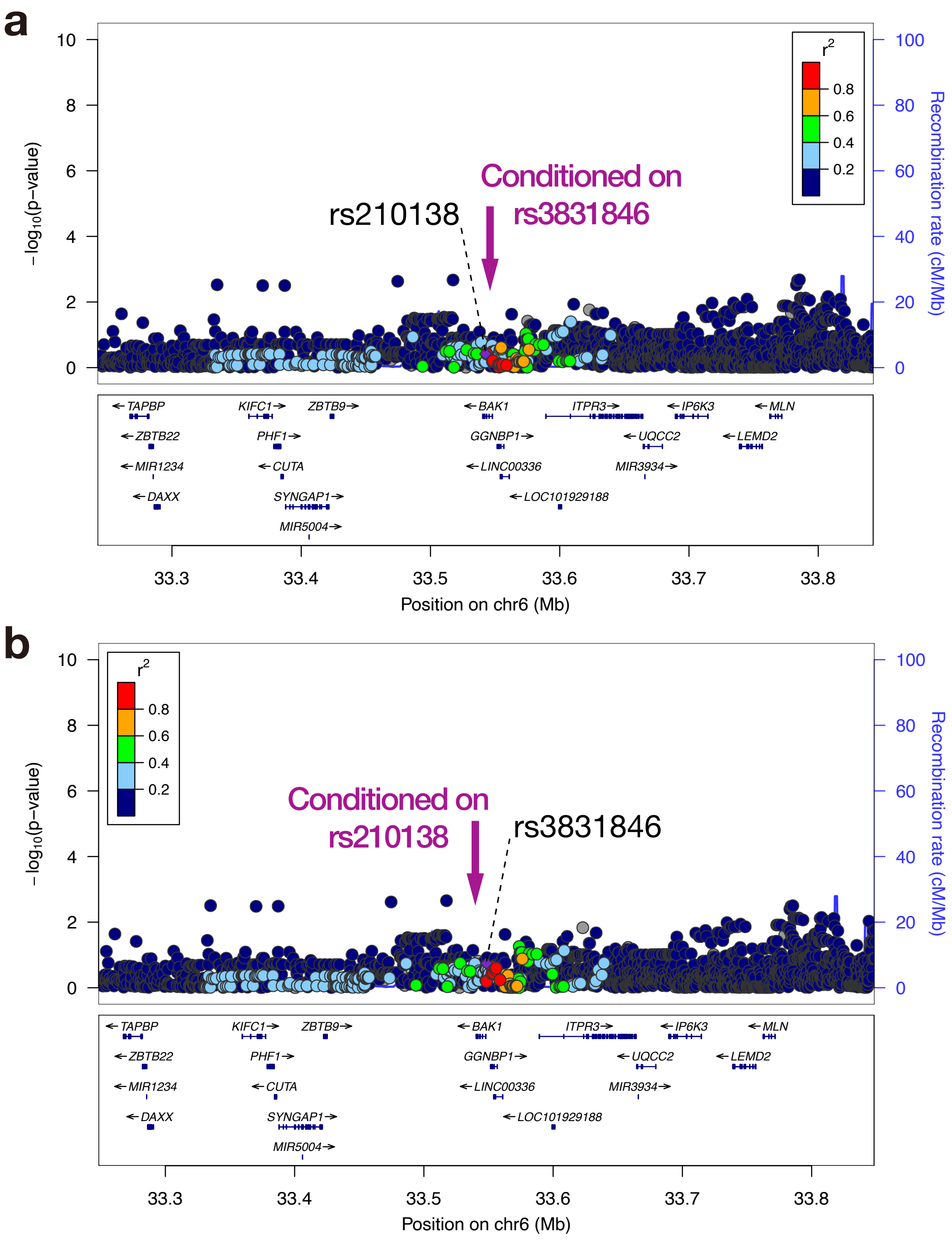

Supplementary Figure 3 | Conditioning analysis performed separately for rs3831846 and rs210138

Regional associations of the imputed genetic variants are shown. **(a)** The significance of variants was evaluated conditioned on the genotype of rs3831846. **(b)** The significance of variants was evaluated conditioned on the genotype of rs210138.

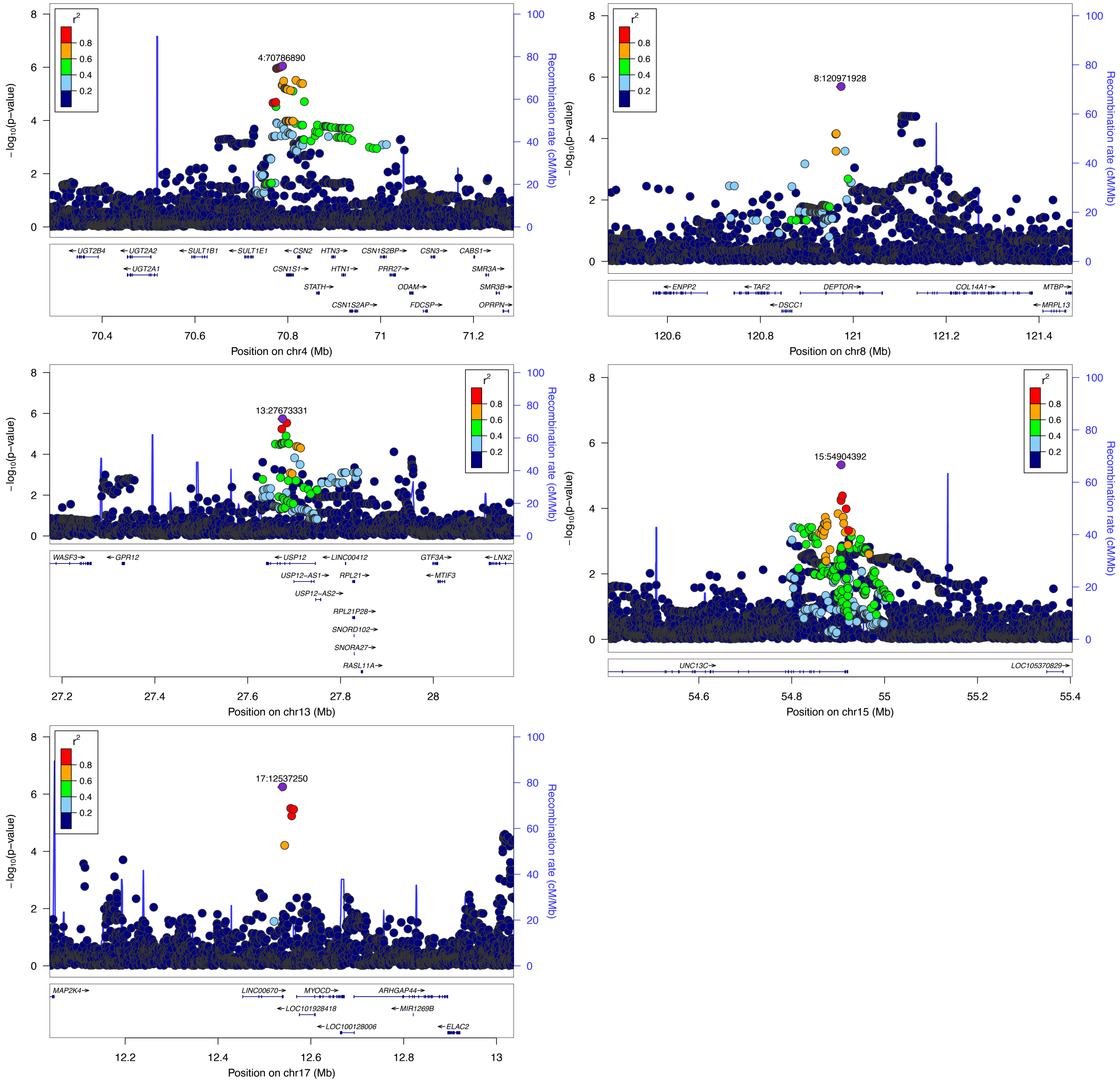

Supplementary Figure 4 | Regional plots of additional five loci with suggestive significance

Regional associations of the imputed genetic variants are shown for each locus with suggestive significance. Purple diamonds indicate lead variants. Other circles are colored by LD (r^2) with the lead variant based on the East Asian individuals of the reference panel used for genotype imputation.

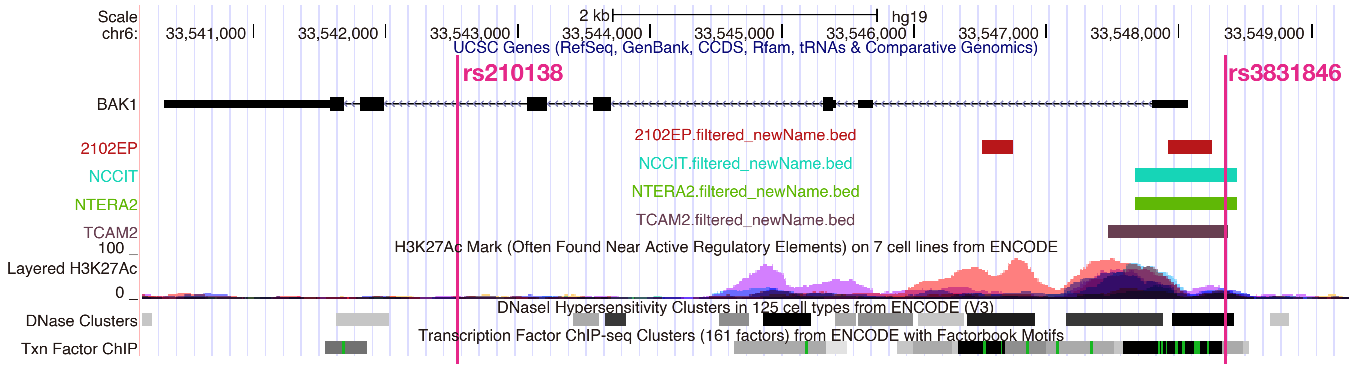

Supplementary Figure 5 | Open chromatin regions at the *BAK1* locus in TGCT cell lines

Open chromatin regions called by ATAC-seq on four TGCTs cell lines (2102EP, NCCIT, NTERA2, and TCAM2) (Pluta, J. et al. *Nat Commun* 2021) are shown. The genomic coordinates of rs3831846 and rs210138 are indicated by pink vertical lines.

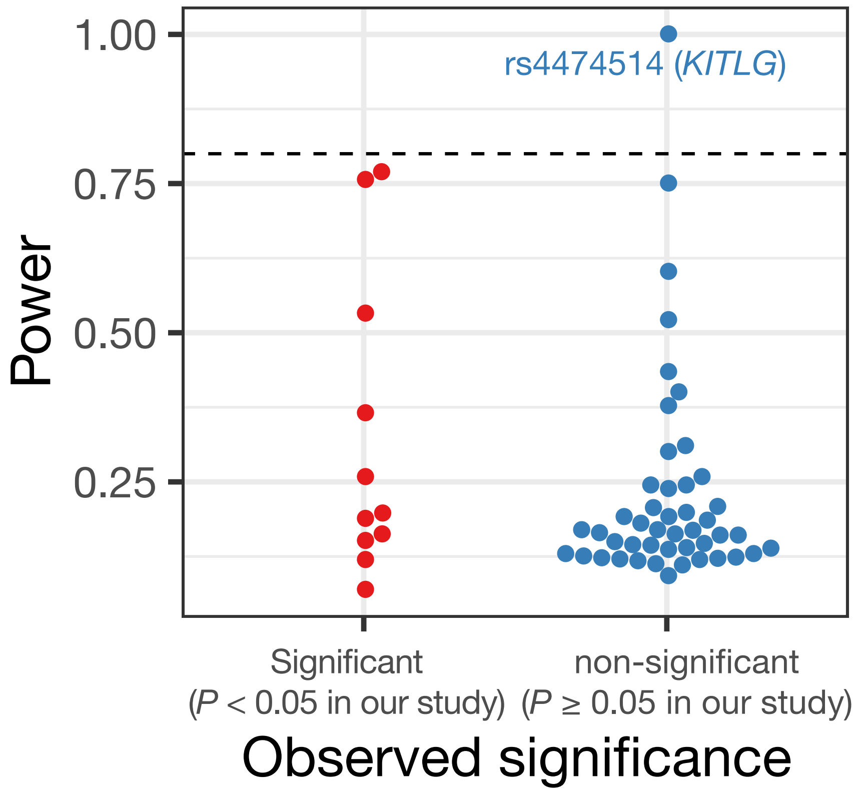

Supplementary Figure 6 | Estimated statistical power for the TGCTs risk variants in the IGCTs GWAS

Each dot represents the statistical power for the TGCTs risk variant. Variants are separately shown for their observed statistical significance in the IGCTs GWAS. Statistical power is calculated based on the odds ratio reported by the European TGCTs GWAS, the allele frequency and sample size in the Japanese IGCTs GWAS data, and the significance threshold of $\alpha = 0.05$. The black dashed line indicates a statistical power of 0.8.

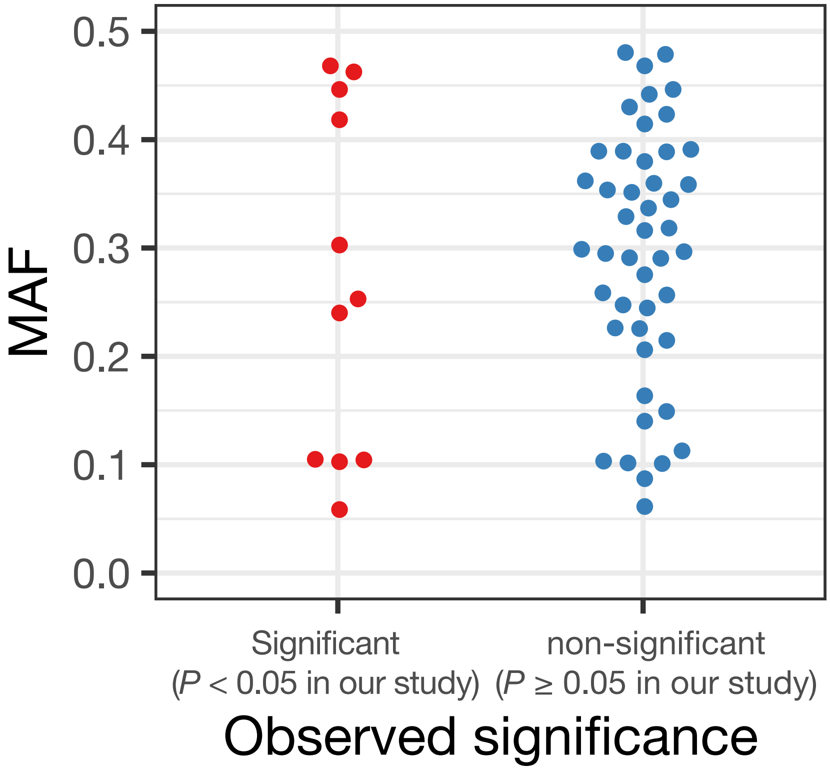

Supplementary Figure 7 | Minor allele frequency of the TGCTs risk variants in the IGCTs GWAS

Each dot represents the minor allele frequency of the TGCTs risk variant in the IGCTs GWAS. Variants are separately shown for their observed statistical significance in the IGCTs GWAS.

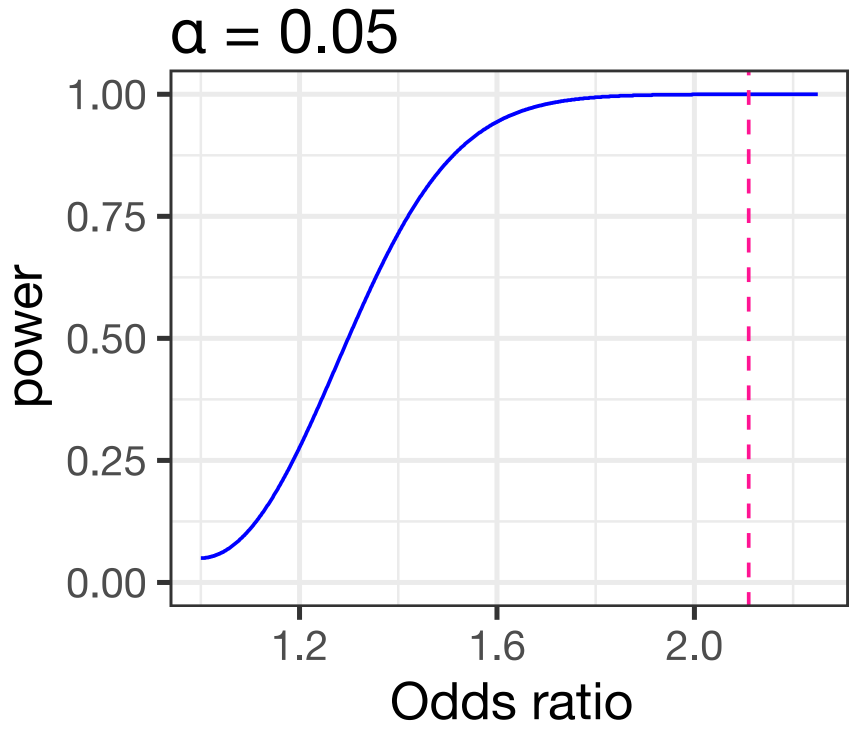

Supplementary Figure 8 | Estimated statistical power for the *KITLG* locus

Statistical power is shown as a function of odds ratio, based on the allele frequency and sample size in the Japanese IGCTs GWAS data and the significance threshold of α = 0.05. The pink dashed line indicates the odds ratio of 2.11 reported in the TGCTs GWAS.

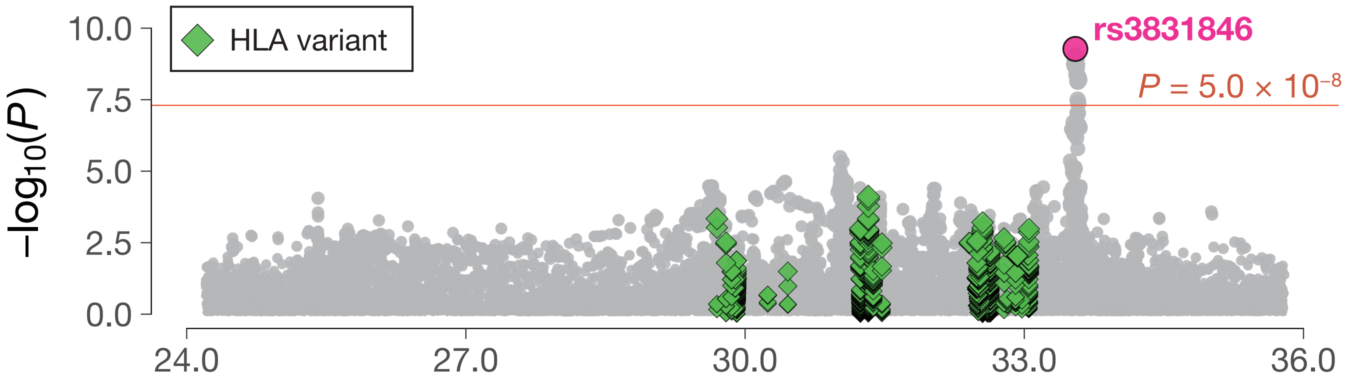

Supplementary Figure 9 | No evidence for the HLA variant associations

Regional associations of the imputed HLA variants and single genetic markers within the major histocompatibility complex region are shown. The associations were tested using a logistic regression model adjusted for the same covariates as the genome-wide association test. Green diamonds indicate two-digit and four-digit HLA alleles and amino acid polymorphisms of the HLA genes. Pink circles indicate the lead variant rs3831846. The orange horizontal lines indicate the genome-wide significance threshold of P value = 5.0×10^{-8}. All statistical tests are two-sided and not adjusted for multiple comparisons.

Supplementary Tables

Supplementary Table 1 | Association results for the lead variants of the suggestive significant loci

Cytoband	SNP	Chr	Position (NCBI build 37)	Alleles	Risk Allele	Freq. Case	Freq. Ctrl	Odds Ratio (95% CI)	P value	Nearest gene
4q13	rs79930945	4	70,786,890	AG/A	A	0.18	0.084	2.72 (1.82–4.05)	9.2×10^{-7}	*SULT1E* *CSN1S1*
8q24	rs56361736	8	120,971,928	A/C	C	0.13	0.052	3.28 (2.01–5.34)	2.1×10^{-6}	*DEPTOR*
13q12	rs4769541	13	27,673,331	G/A	G	0.23	0.13	2.30 (1.63–3.24)	1.9×10^{-6}	*USP12*
15q21	rs1851006	15	54,904,392	A/G	A	0.55	0.40	1.95 (1.47–2.60)	4.7×10^{-6}	*UNC13C*
17p12	rs193269265	17	12,537,250	G/T	T	0.11	0.042	3.67 (2.21–6.11)	5.7×10^{-7}	*LINC00670*

Freq. Case, risk allele frequency in cases; Freq. Ctrl, risk allele frequency in controls. All statistical tests are two-sided and not adjusted for multiple comparisons.

Supplementary Table 2 | Association test between rs3831846 and somatic mutational profiles

Somatic mutation		rs3831846 genotype					*P* value
		TGTAA/TGTAA	TGTAA/T	T/T	Sum	Risk allele frequency	
KIT gene	(+)	4	12	13	29	0.66	0.52
	(-)	13	40	31	84	0.61	
Sum		17	52	44	113		

Somatic mutation		rs3831846 genotype					*P* value
		TGTAA/TGTAA	TGTAA/T	T/T	Sum	Risk allele frequency	
MAPK pathway	(+)	8	21	20	49	0.62	0.93
	(-)	9	31	24	64	0.62	
Sum		17	52	44	113		

Somatic mutation		rs3831846 genotype					*P* value
		TGTAA/TGTAA	TGTAA/T	T/T	Sum	Risk allele frequency	
PI3K pathway	(+)	1	4	2	7	0.57	0.71
	(-)	16	48	42	106	0.62	
Sum		17	52	44	113		

The MAPK pathway includes somatic mutations of *KIT*, *KRAS*, *NRAS*, *HRAS*, *RRAS2*, *NF1*, *CBL*, *FGD6*, *FGFR2*, *TRAF6*, and *F2R*.

The PI3K pathway includes somatic mutations of *MTOR*, *PTEN*, *PIK3C2B*, and *PIK3R2*.

P values were provided by the two-sided Cochran–Armitage trend test.
