# Supplementary material for: A common deletion at BAK1 reduces enhancer activity and confers risk of intracranial germ cell tumors
Source: Nat Commun. 2022 Aug 2;13:4478. doi: 10.1038/s41467-022-32005-9 (PMC9346128; doi:10.1038/s41467-022-32005-9)
Supplement: Supplementary file 2 — Description of Additional Supplementary Files [file 41467_2022_32005_MOESM2_ESM.pdf]

Description of Additional Supplementary Files

File Name: Supplementary Data 1

Description: Comparison of the effect of the TGCTs risk variants between IGCTs and TGCTs

File Name: Source Data

Description: Raw numerical data of Figure 2c.
