# Supplementary material for: A common deletion at BAK1 reduces enhancer activity and confers risk of intracranial germ cell tumors
Source: Nat Commun. 2022 Aug 2;13:4478. doi: 10.1038/s41467-022-32005-9 (PMC9346128; doi:10.1038/s41467-022-32005-9)
Supplement: Supplementary file 4 — Reporting Summary [file 41467_2022_32005_MOESM4_ESM.pdf]

nature portfolio

Corresponding author(s): Yukinori Okada

Last updated by author(s): Jun 13, 2022

Reporting Summary

Nature Portfolio wishes to improve the reproducibility of the work that we publish. This form provides structure for consistency and transparency in reporting. For further information on Nature Portfolio policies, see our Editorial Policies and the Editorial Policy Checklist.

Statistics

For all statistical analyses, confirm that the following items are present in the figure legend, table legend, main text, or Methods section.

n/a	Confirmed	
☐	☒	The exact sample size (*n*) for each experimental group/condition, given as a discrete number and unit of measurement
☐	☒	A statement on whether measurements were taken from distinct samples or whether the same sample was measured repeatedly
☐	☒	The statistical test(s) used AND whether they are one- or two-sided<br>*Only common tests should be described solely by name; describe more complex techniques in the Methods section.*
☐	☒	A description of all covariates tested
☐	☒	A description of any assumptions or corrections, such as tests of normality and adjustment for multiple comparisons
☐	☒	A full description of the statistical parameters including central tendency (e.g. means) or other basic estimates (e.g. regression coefficient) AND variation (e.g. standard deviation) or associated estimates of uncertainty (e.g. confidence intervals)
☐	☒	For null hypothesis testing, the test statistic (e.g. *F*, *t*, *r*) with confidence intervals, effect sizes, degrees of freedom and *P* value noted<br>*Give P values as exact values whenever suitable.*
☒	☐	For Bayesian analysis, information on the choice of priors and Markov chain Monte Carlo settings
☒	☐	For hierarchical and complex designs, identification of the appropriate level for tests and full reporting of outcomes
☐	☒	Estimates of effect sizes (e.g. Cohen's *d*, Pearson's *r*), indicating how they were calculated

Our web collection on statistics for biologists contains articles on many of the points above.

Software and code

Policy information about availability of computer code

Data collection	No software was used for data collection.
Data analysis	GenomeStudio 2.0.4, R version 3.5.3, PLINK 1.90b4.4 and 2.00a3LM, EIGENSOFT version 6.1.4 , shapeit version 2.r904, Minimac3 version 2.0.1, METASOFT version 2.0.0, and PWMScan version 1.1.9

For manuscripts utilizing custom algorithms or software that are central to the research but not yet described in published literature, software must be made available to editors and reviewers. We strongly encourage code deposition in a community repository (e.g. GitHub). See the Nature Portfolio guidelines for submitting code & software for further information.

Data

Policy information about availability of data

All manuscripts must include a data availability statement. This statement should provide the following information, where applicable:

- Accession codes, unique identifiers, or web links for publicly available datasets
- A description of any restrictions on data availability
- For clinical datasets or third party data, please ensure that the statement adheres to our policy

The GWAS summary statistics will be available from the National Bioscience Database Center (NBDC) Human Database (https://humandbs.biosciencedbc.jp/en/) with the accession number of hum0197. Data can also be browsed at our pheweb.jp website [https://pheweb.jp/].
GTEx v8 data: dbGaP study accession phs000424.v8.p2
The position weight matrix (PWM) library of vertebrates from JASPAR 2020: https://jaspar2020.genereg.net/download/data/2020/CORE/JASPAR2020_CORE_vertebrates_redundant_pfms_meme.zip

Field-specific reporting

Please select the one below that is the best fit for your research. If you are not sure, read the appropriate sections before making your selection.

☒ Life sciences ☐ Behavioural & social sciences ☐ Ecological, evolutionary & environmental sciences

For a reference copy of the document with all sections, see nature.com/documents/nr-reporting-summary-flat.pdf

Life sciences study design

All studies must disclose on these points even when the disclosure is negative.

Sample size	This case-control study includes 138 patients with intracranial germ cell tumors (IGCTs) and 808 healthy volunteers. The number of cases is the largest ever reported on IGCTs genetics.<br>For in vitro assays, we determined sample sizes based on our previous experience and the standards commonly used in comparable publications in the field.
Data exclusions	We excluded samples meeting any of the following criteria: genotyping call rate < 97%, closely related to other samples (PI_HAT > 0.17), or of non-East Asian ancestry.
Replication	We validated the genome-wide significant variant detected in the discovery GWAS using an independent collection of IGCTs cases (n = 99) and a control data of a general Japanese population collected by the BioBank Japan Project (n = 1026). The association was replicated with P = 1.7×10^-7.<br>For in vitro assays, each experiment involved three independent experiments in three biological replicates. All attempts of replication were successful and gave similar results.
Randomization	Not applicable due to case-control study design.
Blinding	Not applicable due to case-control study design.

Reporting for specific materials, systems and methods

We require information from authors about some types of materials, experimental systems and methods used in many studies. Here, indicate whether each material, system or method listed is relevant to your study. If you are not sure if a list item applies to your research, read the appropriate section before selecting a response.

Materials & experimental systems

n/a	Involved in the study	
☒	☐	Antibodies
☐	☒	Eukaryotic cell lines
☒	☐	Palaeontology and archaeology
☒	☐	Animals and other organisms
☐	☒	Human research participants
☒	☐	Clinical data
☒	☐	Dual use research of concern

Methods

n/a	Involved in the study	
☒	☐	ChIP-seq
☒	☐	Flow cytometry
☒	☐	MRI-based neuroimaging

Eukaryotic cell lines

Policy information about cell lines

Cell line source(s)	293T cells were purchased from ATCC.
Authentication	As stated in the method, 293T cells used in this study were purchased from ATCC with authentication. These cells were used under 15 passages for preventing genotypic and phenotypic drift and authenticated using morphology / phenotypes with careful monitoring by our lab.
Mycoplasma contamination	The cell line were tested negative for mycoplasma contamination.
Commonly misidentified lines<br>(See ICLAC register)	No commonly misidentified cell line have been used in this study.

Human research participants

Policy information about studies involving human research participants

Population characteristics	All case participants had a primary diagnosis of intracranial germ cell tumors including germinoma, embryonal carcinoma, yolk sac tumor, choriocarcinoma, teratoma, and mixed germ cell tumors in the central nervous system. All case participants are of Japanese ancestry, 17% females and 83% males. Of the 762 controls that passed quality control criteria, 758 (99.5%) were older than the median age at diagnosis of the cases (i.e., 16 years old).<br>Population characteristics of the GTEx dataset is described in the following article:<br>Aguet, F., et al. The GTEx Consortium atlas of genetic regulatory effects across human tissues. Science, 369(6509), 1318-1330 (2020). doi: 10.1126/science.aaz1776
Recruitment	Children and adults diagnosed with intracranial germ cell tumors were identified at the National Center for Child Health and Development and seven other recruiting hospitals throughout Japan and invited to participate in this study. Healthy volunteers were recruited as controls from the Osaka University Graduate School of Medicine, the University of Tokyo, and affiliated institutes. The control group also included genomic DNA from Epstein-Barr virus-transformed B-lymphoblast cell lines of unrelated Japanese individuals established by the Japan Biological Informatics Consortium. To account for population stratification, we incorporated principal components into the regression model as covariates. Although some of the control participants of the discovery dataset are engaged voluntarily, since our main finding is confirmed by the replication dataset that consists only of samples collected during hospital visits, we consider it unlikely that self-selection bias could substantially affect our results.
Ethics oversight	This study was approved by the ethical committee of the National Center for Child Health and Development and Osaka University.

Note that full information on the approval of the study protocol must also be provided in the manuscript.
